# Dynamics of Seed-Borne Rice Endophytes on Early Plant Growth Stages

**DOI:** 10.1371/journal.pone.0030438

**Published:** 2012-02-17

**Authors:** Pablo R. Hardoim, Cristiane C. P. Hardoim, Leonard S. van Overbeek, Jan Dirk van Elsas

**Affiliations:** 1 Department of Microbial Ecology, University of Groningen, Centre for Ecological and Evolutionary Studies, Groningen, The Netherlands; 2 Plant Research International, Wageningen, The Netherlands; Pacific Northwest National Laboratory, United States of America

## Abstract

Bacterial endophytes are ubiquitous to virtually all terrestrial plants. With the increasing appreciation of studies that unravel the mutualistic interactions between plant and microbes, we increasingly value the beneficial functions of endophytes that improve plant growth and development. However, still little is known on the source of established endophytes as well as on how plants select specific microbial communities to establish associations. Here, we used cultivation-dependent and -independent approaches to assess the endophytic bacterrial community of surface-sterilized rice seeds, encompassing two consecutive rice generations. We isolated members of nine bacterial genera. In particular, organisms affiliated with *Stenotrophomonas maltophilia* and *Ochrobactrum* spp. were isolated from both seed generations. PCR-based denaturing gradient gel electrophoresis (PCR-DGGE) of seed-extracted DNA revealed that approximately 45% of the bacterial community from the first seed generation was found in the second generation as well. In addition, we set up a greenhouse experiment to investigate abiotic and biotic factors influencing the endophytic bacterial community structure. PCR-DGGE profiles performed with DNA extracted from different plant parts showed that soil type is a major effector of the bacterial endophytes. Rice plants cultivated in neutral-pH soil favoured the growth of seed-borne *Pseudomonas oryzihabitans* and *Rhizobium radiobacter*, whereas *Enterobacter*-like and *Dyella ginsengisoli* were dominant in plants cultivated in low-pH soil. The seed-borne *Stenotrophomonas maltophilia* was the only conspicuous bacterial endophyte found in plants cultivated in both soils. Several members of the endophytic community originating from seeds were observed in the rhizosphere and surrounding soils. Their impact on the soil community is further discussed.

## Introduction

Endophytes can be defined as microbial communities (bacteria and fungi) that are found inside plant tissues without causing any apparent harm to the host. Microbial endophytes have been reported to occur in virtually all tissues of the host plant, including aseptically regenerated meristematic tissues of micropropagated plants [1], [2]. The concept that seeds may serve as the sources of endophytes or pathogens was first launched by Baker et al. [3]. The presence of bacterial endophytes in, and dissemination from, seeds may be considered to represent an atypical event, which is certainly very difficult to demonstrate. However, the presence of bacteria has been documented in ovule tissues (several plants [4]), throughout seed maturing stages of rice [5] and in the endosphere of mature rice seeds [6]. Still, the concept of seeds as important sources of bacterial endophytes has been called controversial until recently [7]. A recent study revealed that a diverse array of endophytes could be obtained from plant tissue that once was considered germ-free, i.e. the callus tissue of micropropagated plants. This community encompassed a total of 11 bacterial and 17 fungal (ascomycete) taxa [8]. Moreover, a core set of seed-borne endophytes has been demonstrated to endure for hundreds of seed generations, suggesting that select endophytes might establish long relationship with their host thus defeating the boundaries of evolution, human selection and ecology [9]. More recently, the function of seed-borne endophytes that improve seedling development have been demonstrated in a study in which seed-borne *Pseudomonas* sp. SENDO 2, *Acinetobacter* sp. SENDO 1, and *Bacillus* sp. SENDO 6 improved cardon cactus growth by solubilising rock minerals [10]. These results suggest that bacterial endophytes are inherent to plant tissues and may exert more essential functions than is apparent first sight.

The bacterial community inside a plant is obviously prone to influences caused by changing plant physiology [11]. Therefore, many factors that modify plant physiology, e.g. growth stage, soil type, agricultural management regime and even bacterial density, are thought to also promote significant shifts in the endophytic community structure. On the other hand, so-called competent endophytes might thrive in the plant even under adverse conditions [12]. We coined the term ‘competent endophyte’ for microorganism that successfully colonizes the plant tissues and that has the capacity to incite plant physiology and be selectively favoured, leading to beneficial maintenance of the plant-microbe association [13]. For the great majority of bacterial endophytes, their function or ecology inside the host plant is unknown. However, particular bacterial endophytes might actively influence the physiology of the host as a result of the production of phytohormones and/or the modulation of host ethylene levels. Many other plant-growth-promoting functions, such as fixation of N_2_, solubilisation of inorganic phosphate, provision of micronutrients, promotion of photosynthetic activity, induction of the plant defence system, production of antibiotics, biotransformation of heavy metals and biodegradation of organic pollutants, might also enhance host fitness [14]. The effect of these beneficial functions might be drastically improved when plant endophytes establish synergistic interactions with their plant hosts [15]–[17].

In this study we present a comprehensive analysis of the bacterial endophytes of rice seeds by assessing the culture-dependent and -independent fractions of the bacterial community in two consecutive seed generations. Furthermore, we assessed the development of bacterial endophytes from second-generation seeds up to tiller stage of plants growing in gamma-irradiated soils. To gain insight into how environmental factors affect the bacterial endophytic community, we included different abiotic conditions, i.e. we used two soil types (neutral and low pH) and two water regimes (flooded and unflooded). We also assessed different biotic parameters, i.e. we introduced previously isolated bacterial root endophytes in two densities (low and high bacterial inoculation densities - BID) and compared these with an uninoculated treatment. We then assessed the bacterial communities that emerged in the bulk and rhizosphere soils, and in the root and shoot endosphere. We found that the seed-borne bacterial endophytes were highly diverse. As the plant developed, few of these became dominant while others were suppressed. The endophytic community in plant tissue was largely influenced by soil type, followed by water regime. These results suggest that, under our conditions of reduced soil microbial complexity, rice seeds are important sources of bacterial endophytes that colonize the plant. Furthermore, plant physiology was found to play a major role in shaping the structure and diversity of the endophytic bacterial communities.

## Results

### Rice seed endophytic communities

The culturable endophytic community of rice seeds was assessed using the seeds from two consecutive generations. Seeds from the first generation showed the highest population density, at 3.5 10^5^ CFU g^−1^ fresh weight (FW), whereas the second generation revealed the presence of 4.5 10^3^ CFU g^−1^ FW. A total of 16 strains were isolated from internal seed tissues of rice. The 16S rRNA gene identification of these revealed that the endophytes encompassed members of nine genera within the classes *Alpha*- and *Gamma-proteobacteria, Flavobacteria, Bacilli* and *Actinobacteria* (Table 1). Strains that were closely related to *Stenotrophomonas maltophilia* (R2 and R8), *Mycobacterium abscessus* (R1 and R5) and *Ochrobactrum* spp. (R3 – *O. tritici* and R12 – *O. grignonense*) were observed inside both seed generations. The seed endosphere strains R6, R8, R9, R11, R12, R15 and R16 showed high 16S rRNA gene sequence similarities (>99.0%) to bacteria isolated and/or sequenced from the rice phytosphere, rhizosphere and paddy soil (Table 1), suggesting that these bacteria might be well adapt to rice niche.

**Table 1:** Identification of isolated seed-borne strains.

Strains^a^	Accession number	Closest type strain (accession number)	Similarity (%)	Closest rice associated bacteria (accession number)	Similarity (%)	Sources^b^
**R6** *	JN110435	*Pseudomonas protegens* CHA0^T^ (AJ278812)	723/723 (100)	*Pseudomonas* sp. MDR7 (AM911672)	723/723 (100)	R
**R2**	JN110431	*Stenotrophomonas maltophilia* IAM 12423^T^ (AB294553)	789/792 (99.6)	Uncultured *Stenotrophomonas* clone SHCB1148	785/792 (99.1)	RE1
**R8** *	JN110437	*Stenotrophomonas maltophilia* IAM 12423^T^ (AB294553)	662/663 (99.8)	Uncultured *Stenotrophomonas* clone SHCB1148	661/663 (99.7)	RE1
**R3**	JN110432	*Ochrobactrum tritici* SCII 24^T^ (AM114402)	741/741(100)	*Ochrobactrum* sp. RFNB9 (FJ266319)	727/741 (98.1)	PF
**R12**	JN110441	*Ochrobactrum grignonense* OgA9a^T^ (AJ242581)	754/755 (99.9)	*Ochrobactrum* sp. RFNB9 (FJ266319)	749/755 (99.2)	PF
**R7**	JN110436	*Sphingomonas yanoikuyae* IFO 15102^T^ (D13728)	717/721 (99.4)	Uncultured *Sphingomonas* clone SHCB0924	696/723 (96.3)	RE1
**R11**	JN110440	*Flavobacterium johnsoniae* DSM 2064^T^ (AM230489)	608/619 (98.2)	*Flavobacterium* sp. P-135 (AM412169)	615/620 (99.2)	PS
**R4**	JN110433	*Paenibacillus humicus* PC-147^T^ (AM411528)	547/590 (92.7)	*Paenibacillus* sp. RFNB4 (FJ266315)	542/588 (92.2)	PF
**R10**	JN110439	*Agromyces mediolanus* DSM 20152^T^ (X77449)	674/674 (100)	*Curtobacterium* sp. Pd-E-(s)-l-D-6(4) (AB242985)	198/204 (97.1)	SE
**R9**	JN110438	*Curtobacterium citreum* DSM 20528^T^ (NR_026156)	720/721 (99.8)	*Curtobacterium* sp. Pd-E-(l)-e-D-1(4) (AB291847)	203/203 (100)	LE
**R16**	JN110445	*Curtobacterium herbarum* DSM 14013^T^ (AM410692)	798/800 (99.7)	*Curtobacterium* sp. Pd-S-(l)-l-D-3(6) (AB291903)	248/250 (99.2)	LS
**R14**	JN110443	*Frigoribacterium faeni* DSM 10309^T^ (AM410686)	717/719 (99.7)	*Curtobacterium* sp. Pd-E-(l)-e-D-3(5) (AB291849)	194/199 (97.5)	LE
**R15**	JN110444	*Microbacterium oleivorans* DSM 16091^T^ (AJ698725)	791/797 (99.2)	*Microbacterium* sp. Pd-S-(l)-l-D-6(16) (AB291906)	311/311 (100)	LS
**R1**	JN110430	*Mycobacterium abscessus* CIP 104536^T^ (AY457071)	574/576 (99.6)	*Mycobacterium* sp. Pd-E-(r)-m-D-6(5) (AB291833)	329/343 (95.9)	RE2
**R5**	JN110434	*Mycobacterium abscessus* CIP 104536^T^ (AY457071)	622/623 (99.8)	*Mycobacterium* sp. Pd-E-(r)-m-D-6(5) (AB291833)	308/322 (95.6)	RE2
**R13**	JN110442	*Plantibacter flavus* DSM 14012^T^ (AJ310417)	629/630 (99.8)	*Microbacterium* sp. P-65 (AM411961)	615/631 (97.5)	PS

a Rice strains isolated from first (R1-R4) and second (R5-R16) generation of seeds.

*The 16S rRNA gene sequences of strains R6 and R8 were identical to PCR-DGGE products of the bands 12 and 9, respectively.

b Source of the closest rice associated bacteria, LE – Leaf Endophyte [21]; LS – Leaf surface [21]; PF – Paddy Field (Islam et al., unpublished); PS – Paddy Soil [28]; R - Rhizosphere [25]; RE1 - Root Endosphere [20]; RE2 - Root Endosphere [21] and SE – Seed endophyte [5].

**Figure 1:**
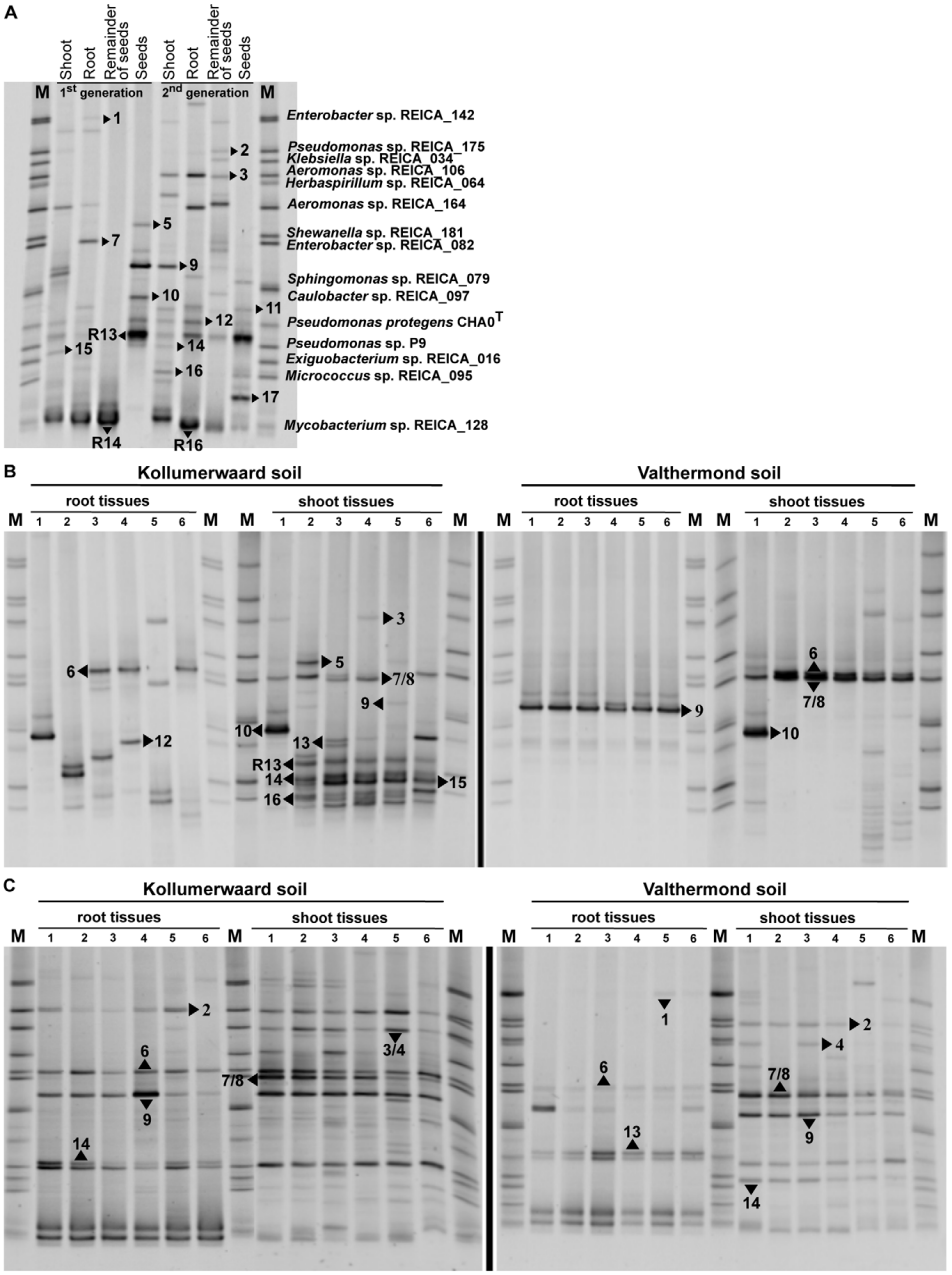
Dynamics of rice endophytes as revealed by PCR-DGGE profiles of seed, three- and five-week-old rice plants. Rice endophyte PCR-DGGE patterns of surface-sterilized dehulled seeds and five-day-old shoot, root and remainder of the seeds from two consecutive generations are shown (panel A). PCR-DGGE patterns of root and shoot endosphere community of three- B) and five- C) week-old rice plants cultivated in two soil types. Six replicates per treatments are shown. Arrow heads indicate identified communities from excised PCR-DGGE bands (only numbers) and strains with identical motility (preceded by letter R; see Table 1 and 2), M – marker with a selection of 15 endophyte ribotypes (panel A).

PCR-DGGE analysis of the seed and rice tissue (5 days) endophytic communities revealed considerable complexity, with a total of 30 migration positions of the bands (Fig. 1A). Across the samples, the bacterial richness varied between 7 and 15 bands, which included five dominant bands (Fig. 1A bands 3, 9, R13, R14 and one as-yet-unidentified band), which were erratically distributed in the midst of many faint ones. Seeds from the first and second generations revealed a similar endophytic richness with, respectively, nine and seven PCR-DGGE bands. Four PCR-DGGE bands (Fig. 1A bands 11, 12, R13 and one as-yet-unidentified band) were shared in both seed generations, whereas three (9, 10 and one as-yet-unidentified, Fig. 1A) were found in the seeds of the first generation and the remainder in the second seed generation. The endophyte richness assessed from shoot and root tissues of aseptically growing rice seedlings showed slightly higher richness than that observed inside seeds with, respectively, 13 and 11 PCR-DGGE bands on average from both generations. The endophytic community that was shared in both generations of seedling shoot and root tissues encompassed, respectively, 24% (PCR-DGGE bands 9, 12, R13, R14 and one as-yet-unidentified) and 22% (bands 11, 12, R13 and one as-yet-unidentified) of the total community.

We tentatively identified 17 PCR-DGGE bands by sequencing (Table 2) and assigned three additional bands with identical motility behaviour to previously isolated seed endophytes (band identity is preceded by letter R, Fig. 1A). In the PCR-DGGE profile of seed and seedling endophytes, a total of 16 PCR-DGGE bands were identified, of which ten showed high 16S rRNA gene sequence similarity (>99.0%) to bacteria previously assessed from the root endosphere of mature rice plants growing in the Philippines (Fig. 1A, PCR-DGGE bands 1, 2, 3, 4, 5, 7, 9, 10 and 14) and from the rhizosphere of rice plants growing in India (Fig. 1A, band 12; Table 2). PCR-DGGE bands 9, 12 and R13 were the most frequently found bands inside seeds and seedlings of both generations. They were closely related to *S. maltophilia* (99.7% sequence similarity), *Pseudomonas protegens* CHA0^T^ (100%) and *Plantibacter flavus* DSM 14012^T^ (99.8%), respectively (Tables 1 and 2). The bands of seed endophyte strains R6 and R8 showed migration behaviour similar to those of PCR-DGGE bands 12 and 9 and were identical 16S rRNA gene sequence, respectively. Two PCR-DGGE bands with identical motility (3 and 4, and 7 and 8) were identified as belonging to different species and these were further analysed as pairs.

**Table 2:** Identification of excised PCR-DGGE bands.

DGGE band ID	Accession number	Closest type strain or known strain (accession number)	Similarity (%)	Closest rice associated bacteria (accession number)	Similarity (%)	Sources^a^
**1**	JN110446	*Enterobacter cloacae* subsp. *cloacae* ATCC 13047^T^ (AJ251469)	378/382 (99.0)	*Enterobacter* sp. REICA_142	382/382 (100)	RE1
**2**	JN110447	*Pseudomonas oryzihabitans* IAM 1568^T^ (AM262973)	379/380 (99.7)	*Pseudomonas* sp. REICA_175	379/380 (99.7)	RE1
**3**	JN110448	*Aeromonas hydrophila* subsp. *dhakensis* LMG 19562^T^ (AJ508765)	371/373 (99.5)	*Aeromonas* sp. REICA_106	373/373 (100)	RE1
**4**	JN110449	*Herbaspirillum rubrisubalvicans* ICMP 5777^T^ (AF137508)	346/349 (99.1)	*Herbaspirillum* sp. REICA_064	346/349 (99.1)	RE1
**5**	JN110450	*Acinetobacter beijerinckii* LUH 4759^T^ (AJ626712)	382/382 (100)	Uncultured *Acinetobacter* clone SHCB0621	381/382 (99.7)	RE1
**6**	JN110451	*Rhizobium radiobacter* IAM 12048^T^ (AB247615)	378/383 (98.7)	Uncultured *Rhizobium* SHCB0425	369/386 (95.6)	RE1
**7**	JN110452	*Enterobacter arachidis* Ah-143^T^ (EU672801)	374/376 (99.5)	*Enterobacter* sp. REICA_082	376/376 (100)	RE1
**8**	JN110453	*Escherichia coli* O111:H str. 11128 (AP010960)	382/382 (100)	*Enterobacter* sp. REICA_128	378/382 (98.9)	RE1
**9**	JN110454	*Stenotrophomonas maltophilia* IAM 12423^T^ (AB294553)	382/383 (99.7)	Uncultured *Stenotrophomonas* SHCB1148	382/383 (99.7)	RE1
**10**	JN110455	*Pantoea agglomerans* DSM3493^T^ (AJ233423)	380/380 (100)	Uncultured *Pantoea* SHCB0588	378/380 (99.5)	RE1
**11**	JN110456	*Neisseria meningitidis* M01-240149 (CP002421)	374/375 (99.7)	Uncultured bacterium clone J-3FECA52 (DQ340883)	291/308 (94.5)	RE2
**12**	JN110457	*Pseudomonas protegens* CHA0^T^ (AJ278812)	378/378 (100)	*Pseudomonas* sp. MDR7 (AM911672)	378/378 (100)	R
**13**	JN110458	*Dyella ginsengisoli* Gsoil 3046^T^ (AB245367)	373/373 (100)	*Dyella* sp. V-6.1 (JF429979)	367/373 (98.4)	PF
**14**	JN110459	*Pseudomonas putida* BIRD-1 (CP002290)	378/378 (100)	Uncultured *Pseudomonas* SHCB0777	378/378 (100)	RE1
**15**	JN110460	*Bacillus psychrosaccharolyticus* S156^T^ (AY509230)	373/379 (98.4)	*Bacillus* sp. P-150 (AM412171)	367/381 (96.3)	PS
**16**	JN110461	*Deinococcus ficus* CC-FR2-10^T^ (AY941086)	377/379 (99.5)	Uncultured bacterium clone J-3FECC29 (DQ340907)	266/293 (90.8)	RE2
**17**	JN110462	*Achromobacter spanius* LMG 5911^T^ (AY170848)	367/374 (98.1)	Uncultured bacterium clone J-3FECC48 (DQ340912)	365/374 (97.6)	RE2

a Source of the closest rice associated bacteria: PF – Paddy Field [65]; PS – Paddy Soil [28]; R - Rhizosphere [25]; RE1 - Root Endosphere [20] and RE2 - Root Endosphere [64].

We further compared the rice endophytic community against publicly-available endophytic sequences from seeds of rice (*Oryza sativa*) and *Zea* plants. The strains R9, R15 and R16 were closely related to sequences of endophytes that were exclusively found in rice seeds from two independent studies, whereas PCR-DGGE bands 6 and 10 were closely related to strains found in rice and *Zea* seeds (Table 3). The sequences of strains R6 and R8 and of PCR-DGGE bands 2 and 9 were closely related (>99.0% 16S rRNA sequence similarity) to those of endophytic communities found in rice and *Zea* seeds (Table 3).

**Table 3:** Closest match of sequences obtained in this study against public available rice and *Zea* seed endophyte sequences.

Isolate/DGGE band	Rice						Zea	
	Okunishi et al. [19]	Similarity (%)	Mano et al. [5]	Similarity (%)	Liu et al. unpublished	Similarity (%)	Johnston et al. [9]	Similarity (%)
**R2**			*Stenotrophomonas* sp. Pd-S-(s)-e-D-1(4) (AB242927)	301/302 (99.7)			*Stenotrophomonas* sp. DJM1G3 (JF753464)	516/516 (100)
**R6**							*Pseudomonas* sp. DJM1C10 (JF753430)	513/517 (99.2)
**R8**			*Stenotrophomonas* sp. Pd-S-(s)-e-D-1(4) (AB242927)	174/174 (100)			*Stenotrophomonas* sp. DJM1G3 (JF753464)	514/515 (99.8)
**R9**			*Curtobacterium* sp. Pd-E-(s)-l-D-6(4) (AB242985)	241/241 (100)	*Curtobacterium* sp. Fek20 (EU741030)	721/721 (100)		
**R15**	*Microbacterium* sp. S-(s)-l-D-6(20) (AB178212)	405/408 (99.3)			*Microbacterium* sp. Fek04 (EU741023)	796/797 (99.9)		
**R16**			*Curtobacterium* sp. Pd-E-(s)-m-D-4(12) (AB242967)	229/231 (99.1)	*Curtobacterium* sp. Fek20 (EU741030)	795/800 (99.4)		
**band 2**					*Pseudomonas* sp. Fek13 (EU741028)	379/380 (99.7)	*Pseudomonas* sp. DJM1A4 (JF753403)	379/380 (99.7)
**band 6**					*Agrobacterium* sp. FeL02 (EU741035)	377/382 (98.7)	*Rhizobium* sp. DJM1H4 (JF753477)	381/382 (99.7)
**band 9**							Uncultured bacterium clone DJM126 (JF753390)	382/383 (99.7)
**band 10**					*Pantoea* sp. Aek32 (EU741010)	378/380 (99.5)	Uncultured bacterium clone DJM51 (JF753316)	378/380 (99.5)

### Dynamics of rice endophytic communities as revealed by plant development

As evidenced by PCR-DGGE, the endophytic bacterial communities inside root and shoot tissues of three- and five-week-old rice plants cultivated in gamma-irradiated Kollumerwaard (K) and Valthermond (V) soils were mainly influenced by soil type (Fig. 1B and C). The richness of endophytes from plants cultivated in the K soil was higher than that found in V soil plants, independent of the plant tissue or time of analysis. The profile of the endophytic community from three-week-old plants cultivated in K soil showed two to eight bands for root and eight to 13 bands for shoot tissues, whereas plants cultivated in V soil harboured between two and four and three and 13 bands, respectively. Plants cultivated in K soil showed dominance of five bacterial communities (Fig. 1B PCR-DGGE bands 7/8, R13, 14, 15, and 16) across shoot replicates, whereas the community structure from root tissues was erratically distributed across replicates, with members of the dominant shoot community found in a single replicate (Fig. 1B). One PCR-DGGE band (9) was conspicuously present in all root samples of plants cultivated in V soil, whereas two bands (6 and 7/8) were dominant in the shoot tissues (Fig. 1B).

The PCR-DGGE profiles of the endophytic community from five-week-old plants cultivated in K soil showed four to seven bands in root tissues, of which four (bands 2, 6, 9 and 14, Fig. 1C) were conspicuous. In shoot tissues, 12–16 bands were found, of which six (bands 2, 3/4, 6, 7/8, 9 and 14, Fig. 1C) were conspicuous. The PCR-DGGE profile of plants cultivated in V soil showed five to seven bands in the root tissues, of which two (bands 6 and 13) were conspicuous, and six to 11 were found in shoot tissues, from which five (bands 2, 7/8, 9, 13, 14) were conspicuous.

The endophytic bacterial community of three- and five-week-old rice plants revealed high similarity with types found inside seeds and seedlings, with, respectively, 20 out of 24 and 19 out of 22 PCR-DGGE bands (Fig. 1). Comparison of the endophytic communities during plant growth revealed diverse trends. For instance, in plants cultivated in K soil, the PCR-DGGE bands 3/4 and 9 were erratically found inside seedlings and three-week-old plant tissues, but they became dominant in the shoot tissues of five-week-old plants. Band 6 was also dominant in the five-week samples, however it was never found inside seeds. Other PCR-DGGE bands (5, 10, 12, 13, R13, 15 and 16) found inside the seeds were encountered in the three-week-old plants and not in the five-week samples. Others (bands 11, 17, R14, R16) were only found in the seedlings. Plants cultivated in V soil revealed different patterns, with PCR-DGGE bands 9 and 13 being conspicuously found across the replicates of three-week-old plants (only root tissues) and five-week-old plants (in both tissues), whereas band 1 (found in seeds) was erratically found in five-week-old plants (in both tissues). PCR-DGGE bands 2, 3/4, 10, 14 and 16 were exclusively found in shoot tissues (Fig. 1).

### Endophytic bacterial community survey under distinct conditions

To obtain insight into how the endophytic bacterial community in rice evolves in natural conditions, we designed an assay in which we reduced the complexity of the system (i.e. rice growing in gamma-irradiated soil inoculated with ‘artificial’ community encompassed by 18 selected endophytic strains) and then assessed the bacterial community from four distinct microhabitats (i.e. bulk and rhizosphere soil, root and shoot endosphere tissue). In addition to biotic factors, we investigated two abiotic factors, i.e. two soil types (K and V) and two water regimes (flooded and unflooded). As revealed by the PCR-DGGE profiles, soil exerted a major influence on the endophytic bacterial community structure and were analysed separated.

#### Bacterial distribution on K soil

**Figure 2:**
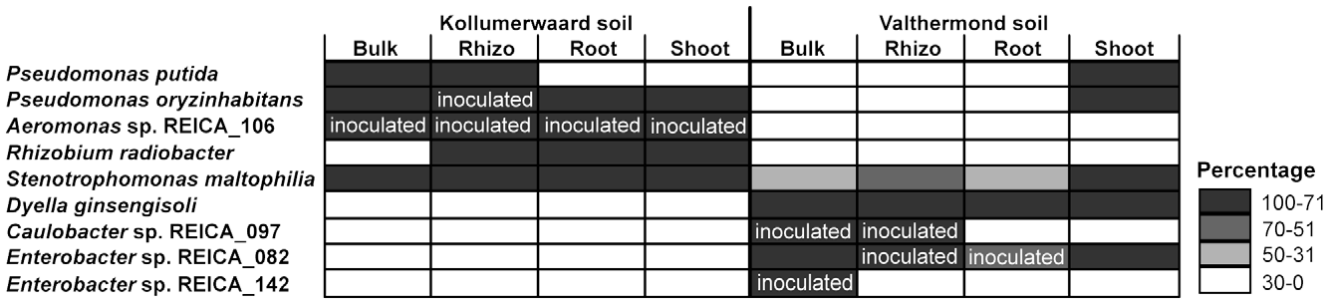
Heat map composition of selected bacterial communities. Distribution of select endophytic bacterial communities (rows) from two soil types (K and V) and four different habitats (root-free and rhizosphere soil, root and shoot endosphere) is shown. Cells are coloured in spectrum of grey that correlates with percentage of observed bacterium in a given habitat. Habitat from which the assessed bacterium was most likely to be originated from ‘artificial’ soil community is labelled with “inoculated”. Unlabelled cells are most likely represented by assessed bacterium originated from rice seeds.

The seed-borne *Pseudomonas oryzihabitans* (PCR-DGGE band 2) and *Stenotrophomonas maltophilia* (band 9) were observed in all analysed habitats of plant cultivated on K soil (Fig. 2; Fig. S1). The introduced *Aeromonas* sp. REICA_106 (band 3) were also observed in all habitats, however only for inoculated treatments, whereas *Rhizobium radiobacter* (band 6) was found in the rhizosphere soil, root and shoot tissues, *Pseudomonas putida* (band 14) was conspicuously found in bulk and rhizosphere soils and *Herbaspirillum* sp. REICA_064 (band 4) was restricted to shoot tissues (Fig. 2).

#### Bacterial distribution on V soil

Plants from V soil selected for members of *Enterobacter* sp. REICA_082 (PCR-DGGE band 7) and *Dyella ginsengisoli* (band 13) for all habitats and *Stenotrophomonas maltophilia* (band 9) mainly in the shoot tissues (Fig. 2; Fig. S2). *Pseudomonas oryzihabitans* (band 2) and *Pseudomonas putida* (band 14) were restricted to shoot tissues, *Enterobacter* sp. REICA_142 (band 1) to bulk soil and the introduced *Caulobacter* sp. REICA_097 to bulk and rhizosphere soils (Fig. 2).

### Factors affecting the endophytic bacterial community composition of rice

**Figure 3:**
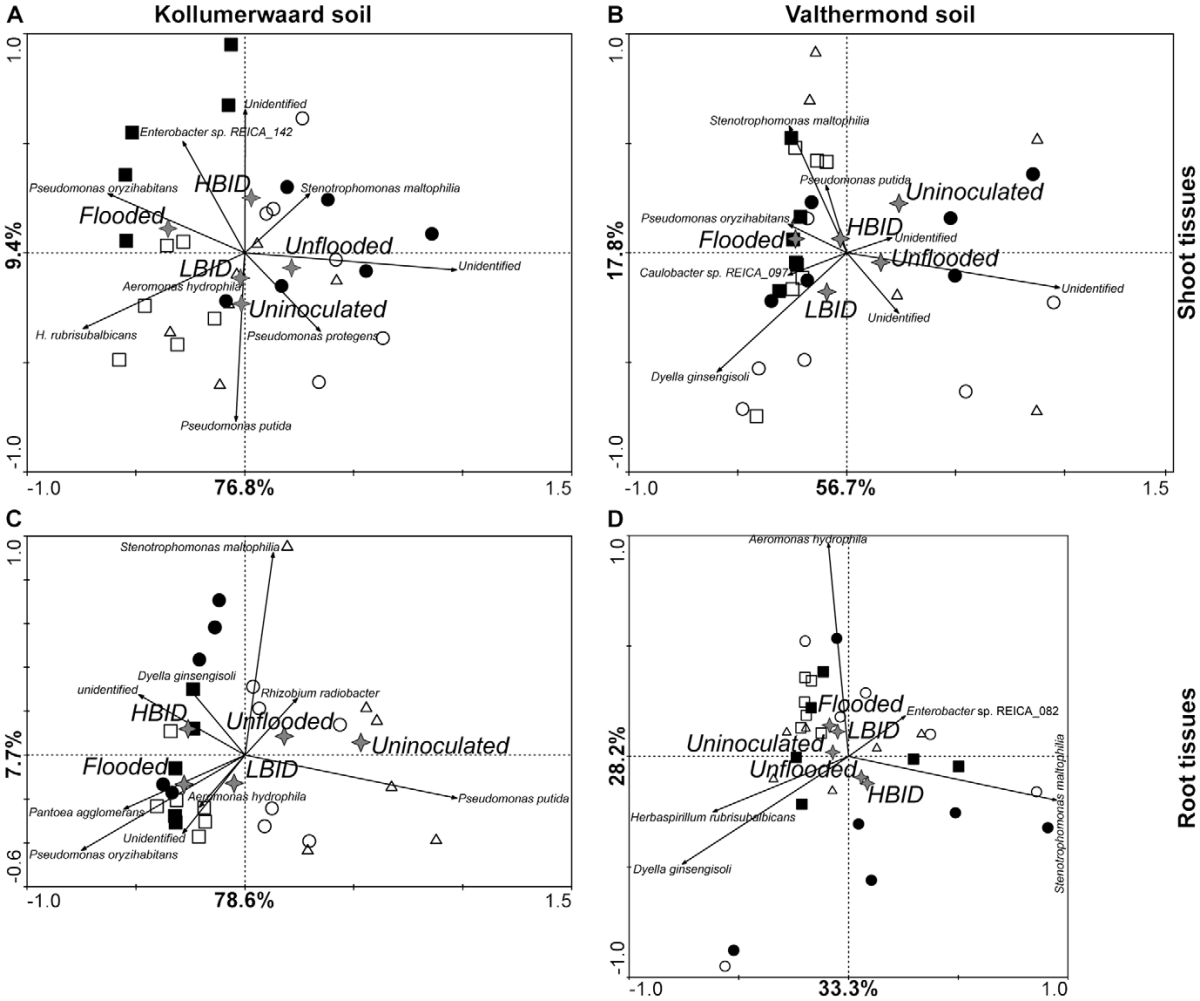
Biplot ordination diagrams of rice shoot and root bacterial endophytes. RDA diagrams generated from PCR-DGGE profiles of endophytic bacterial community sampled from shoot (A and B) and root (C and D) tissues of plants cultivated on K (A and C) and V (B and D) soils are shown. Squares and circle represent PCR-DGGE patterns of bacterial communities from plants submitted to, respectively, flooded and unflooded regimes and exposed to low- (empty symbol) and high- (full symbol) BID. Triangles (control treatment) represent PCR-DGGE patterns of bacterial communities from plants submitted to unflooded regime and cultivated in uninoculated soils. Six replicates of each treatment are shown. Stars represent nominal environmental variables. Arrows represent PCR-DGGE bands in which only the most descriptive communities are shown.

**Figure 4:**
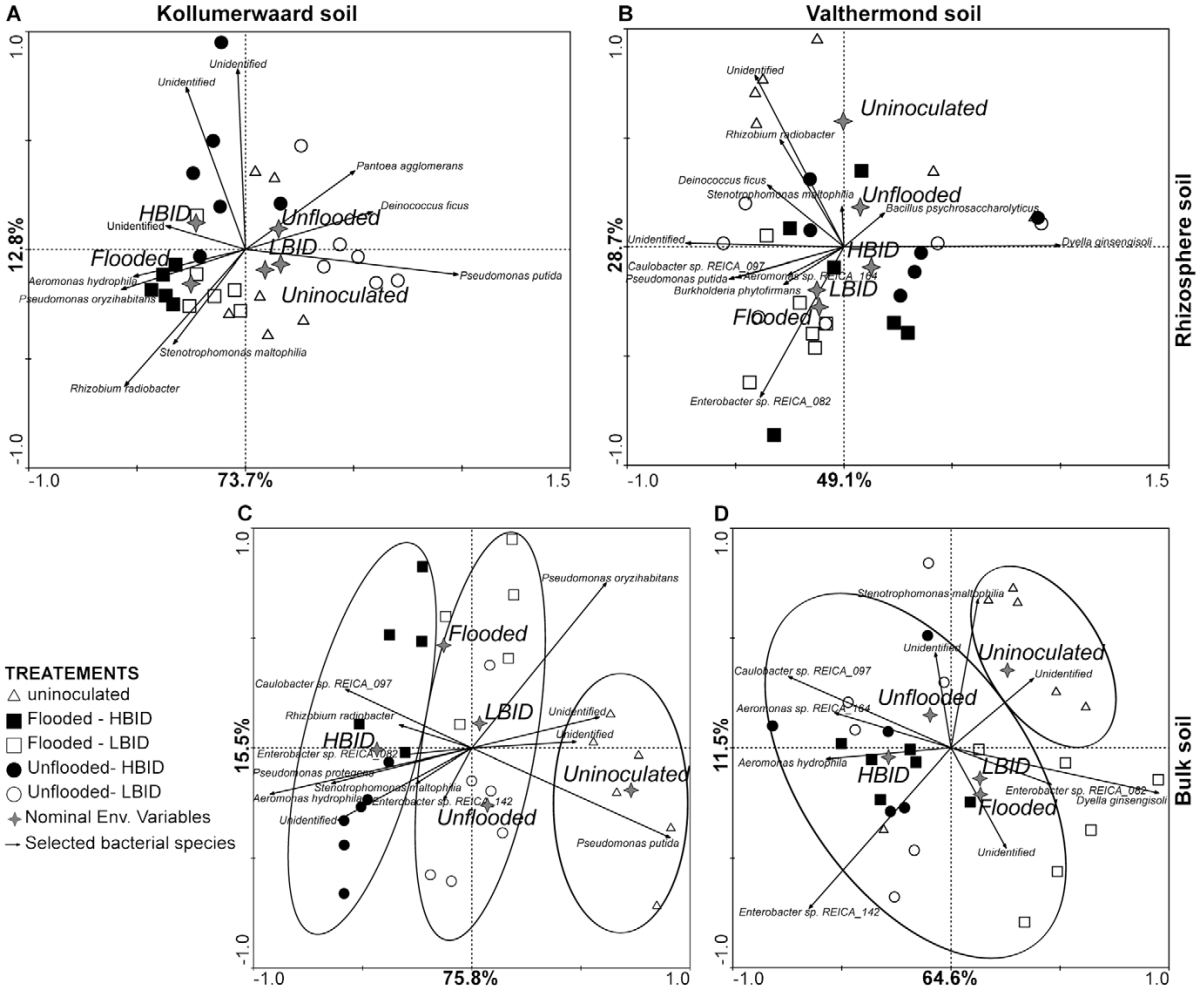
Biplot ordination diagrams of rice rhizosphere and bulk soil bacterial communities. RDA diagrams generated from PCR-DGGE profiles of bacterial community sampled from rhizosphere (A and B) and bulk (C and D) soil of plants cultivated in K (A and C) and V (B and D) soils are shown. See Fig. 3 for symbol description.

Using the collective data, we performed the Redundancy Analysis (RDA) for each habitat separately, per soil type (Fig. 3 and 4). For both soil types the factors affecting the bacterial community composition shifted from water regime treatments in the shoot and root endosphere to the bacterial inoculation densities (BID) on the soil.

#### Distribution of bacterial communities inside shoot tissues

On both soil types the rice shoot endosphytes were mainly influenced by water regimes, where the endosphere community of plants subjected to flooded regime differ significantly from those plants conditioned to unflooded treatment (Fig. 3A and B). A total of 76.6 and 69.2% of the RDA diagram distribution was explained by the water regimes of plants cultivated on K and V soils, respectively. The BID treatments were indistinguishable in the K and V soils and only in the K soil the bacterial community from uninoculated treatments differ significantly from inoculated ones. This suggested that the introduced ‘artificial’ community had exerted a relatively minor effect on the endophytic shoot community for the period investigated.

#### Distribution of bacterial communities inside root tissues

The distribution of root endophere bacterial community differ on both soils. In the K soil, the endophytic community from uninoculated soil differ significantly from plants exposed to high BID. Both treatments explained 50.2% of the total distribution, while water regimes, which also differ significantly, explained 44.6%. The endophytic bacterial communities from root tissues of plants cultivated on uninoculated soil were placed along the second RDA axis, differing from those of plants cultivated in low- and high-BID soil (Fig. 3C). In contrast to K soil, the distribution of root endophytic communities in V soil seems to be indifferent for bacterial inoculation, where plants of uninoculated soil resembled those from plants of inoculated soil (Fig. 3D). However, the root endophytic community of plants cultivated under dissimilar water treatments differed significantly, where plants under flooded and unflooded regimes were separated along the diagonal of the RDA diagram. Around 60% of the total distribution was explained by the abiotic factors.

#### Distribution of bacterial communities in the rhizosphere

As observed on the root tissues, the rhizosphere bacterial communities vary drastically between soil types. In the K soil, most of the treatment was significantly different and the samples from each individual treatment were virtually clustered within one quarter of the RDA diagram. Only the samples from the rhizosphere community of plants cultivated on uninoculated soil were distributed around the centre of the diagram (Fig. 4A). In the V rhizosphere soil, none of the treatments were significantly different, however four out six samples from plants cultivated on uninoculated soil revealed distinct rhizosphere communities and clustered apart from other samples (Fig. 4B).

#### Distribution of bacterial communities on root-free soils

The localization of the soil communities in the RDA diagram was mainly influenced by biotic factors for both soil types. In the K soil, the bacterial communities from high-BID, low-BID and uninoculated soils were distributed along the second RDA axis and differed from each other in three main clusters (Fig. 4D). The biotic factors explained 78.8% of the total diagram distribution, while water regimes counted for 17.4%. In the V soil, three clusters were also detected for each BID and uninoculated treatments. The samples were distributed along the second RDA axis, whereas the bacterial communities from flooded and unflooded regimes were distributed along the first axis (Fig. 4D). The biotic factors explained 53.8% of the total bacterial community distribution on root-free soil, while water regimes counted for 33.8%.

## Discussion

In this paper, we clearly showed that rice seeds are important sources of the endophytic bacteria that come up in the early rice growth stages. This was evidenced by experimentation with plants grown in soils deprived of bacterial communities by irradiation. The contention of seed carriage of key endophytes for young plants was supported by three lines of evidence found in this study.

1. Many (74%) of the rice seed-borne bacterial endophytes found in this study were closely related to bacteria that have previously been isolated from inside maturing and/or mature rice seed tissues [5], [18], [19] and the endosphere of rice root [20] and leaf tissues [21]. Further, they resembled bacteria from the rhizoplane of rice [22], wheat [23] and sacred fig (*Ficus religiosa*) [24], the rhizosphere of rice [25], the phyllosphere of grasses [26] and rice [20], hay dust [27] and soil in which rice had been cultivated [28].
2. Throughout plant development, shoot tissues showed higher bacterial endophyte richness than root tissues. Plants cultivated in open fields often reveal the opposite trend, with higher bacterial richness in the root tissues [29]. Mano et al. [21] observed that the endophytic bacterial community in the leaves of rice plants cultivated in the open field was similar to that found in seed tissues, differing drastically from that inside root tissue. The results suggested that rice seed endophytes are generally adapted to plant tissue and rapidly colonize rice shoots, in which there is less competition than in the respective root, which is bathed in rich bacterial communities.
3. The bacterial community from internal plant tissues and the soil surrounding plant roots (cultivated in soil containing an introduced bacterial community or remaining uninoculated) showed similar endophytic bacterial communities, however they differed in the rhizosphere being unrelated to those in the soil.

The bacterial diversity associated with the rice seeds was actually quite astonishing. Recently, two separate studies investigated the correlation of the bacterial community associated with rice seeds across 12 sampling sites [18] and of those with *Zea* seeds across host genotype (i.e. wild ancestor to domesticated maize) [9]. The studies revealed large diversities (284 genomic fingerprint types determined by BOX-PCR from rice seeds and 26 isolated genera from *Zea* seeds) of the bacterial communities associated with the seeds. However, a great majority of the isolates was correlated to the sampling site where the seeds were derived from or to plant genotype, recapitulating the phylogenetic pattern of their *Zea* hosts. Only a few, such as *Enterobacter cloacae*, *Pseudomonas oryzihabitans* (in both rice and *Zea* seeds), *Curtobacterium* spp. (only in rice seeds), *Clostridium beijerinckii*, *Methylobacterium* sp., *Paenibacillus barcinonensis* and *Pantoea agglomerans* (only in *Zea* seeds) were conserved across the sampling sites and host genotypes [9], [18]. In addition, strains assigned to *Rhizobium radiobacter*, *Stenotrophomonas maltophilia*, *Acinetobacter* spp., *Herbaspirillum rubrisubalbicans* and *Microbacterium* spp., were isolated from rice seeds collected in more than one (but not all) sampling site [18]. These might be also widespread among rice genotypes. In our study, members of *Rhizobium radiobacter*, *Pantoea agglomerans*, *Stenotrophomonas maltophilia*, *Pseudomonas oryzihabitans, Pseudomonas* spp., *Curtobacterium* spp. and *Microbacterium* spp. were also identified. These results suggest that these bacteria are highly adapted to the plant niche.

Many of the aforementioned bacteria are ubiquitous in a range of environment niches, being commonly found in seeds and in the endosphere tissues of rice [5], [18], [19], gramineous (e.g. maize [9]) and leguminous (e.g. soybean [30]) plants, as well as in the soils where these plants had been cultivated. Thus, one might speculate that these organisms form a core microbiota which is conserved across several plant species and that they might use seeds for their own dissemination. For instance, *Stenotrophomonas maltophilia* is an opportunistic bacterium that is often found in soils and in association with plants [31]. It also has a worldwide distribution. Many strains of *Stenotrophomonas maltophilia* have been isolated from the rhizosphere and endosphere of various plants [32]. When inoculated, strains of *Stenotrophomonas* have been shown to enhance plant biomass production in corn [33], sorghum [34], canola [35], potato [36] and poplar [37], all cultivated under greenhouse conditions. Although the genome analysis of *Stenotrophomonas maltophilia* R551-3 has revealed many genes that are dedicated to motility, adaptation to, and colonization of, plant host tissue [38], our results showed that *Stenotrophomonas maltophilia* is transmitted via seeds and can spread out of the host invading the rhizosphere and even surrounding soils. The results suggest that *Stenotrophomonas maltophilia* is highly adapted to niches within the plant and that both dissemination and colonization are two main strategies used in the response to ecological opportunities.

The ecological role of seed endophytes is not thoroughly known. Recently, Puente et al. [10] demonstrated that seed bacterial endophytes are involved in the establishment of giant cardon cactus (*Pachycereus pringlei*) on barren rocks. Cactus seeds disinfected with antibiotics halt seedling development. Plant growth was restored by inoculation of endophytes involved in rock weathering [10]. In another study, introduction of an endophytic consortium composed of *Enterobacter* sp. S_d17, *Pseudomonas* sp. strains S_d12 and S_d13 or of individual strains isolated from surface-sterilized *Nicotiana tabacum* seeds revealed positive effects on plant growth under conditions with and without induced stress (i.e. Cd stress) [39]. The beneficial effects of bacterial endophytes are often more evident in plants cultivated on marginal soils used for phytoremediation or soils conducive to plant disease development [40], [41]. Many seed-borne endophytes are involved in plant growth promotion. This is certainly the case for the conserved seed-borne endophytes *Enterobacter cloacae* and *Pseudomonas oryzihabitans*. For instance, *Enterobacter cloacae* strain 501R3 and other unidentified strain are involved in the suppression of damping-off caused by *Pythium ultimum* in many hosts via competitive colonization of the spermosphere and rhizosphere soils, thus reducing the availability of exuded carbohydrate, lipid and amino acid compounds [42], [43]. In addition, *Enterobacter cloacae* strain UW5 is involved in the production of IAA [44] and the modulation of plant ethylene levels via 1-aminocyclopropane-1-carboxylate (ACC) deaminase [45]. An extensive assessment of the root endophytic community from mature rice plants cultivated in field soil revealed that members of the genus *Enterobacter* were the most abundant and the most genetically diverse isolated bacteria [20]. Although we have not isolated any *Enterobacter* strain in this particular study, we identified two PCR-DGGE bands from first- and second-generation seed profiles that were identical (at 16S rRNA gene sequence level) to the previously found *Enterobacter* members. Both *Enterobacter* sp. strains REICA_142 and REICA_082 revealed plant-growth-promoting properties such as fixation of N_2_, solubilisation of inorganic phosphate and production of ACC deaminase [20]. Members of *Pseudomonas oryzihabitans* containing ACC deaminase (strain Ep4 [46]), or capable of solubilising inorganic phosphate (strain B4M-K [47]), production of IAA, siderophore and fixation of N_2_ (strain G6 [48]) have been reported to increase host biomass. In this study, we identified a member closely related to *Pseudomonas oryzihabitans* that extensively colonized plants cultivated in the neutral-pH soil but was almost absent on roots of plants cultivated in the low-pH soil, suggesting pH sensitivity and possibly the importance of plant physiology for community establishment. In addition, we isolated another species, *Pseudomonas* sp. strain R6, that was closely related to the widespread plant-protecting *Pseudomonas protegens* CHA0^T^, which is capable of producing the antimicrobial compounds 2,4 diacetyl phloroglucinol and pyoluteorin [49]. The results suggested that selected bacterial communities are hosted by seeds, which might become important when differentially beneficial functions are stimulated in accordance with the local conditions. This may support the development of the new host.

Here, the endophytic bacterial community of rice was shown to be largely influenced by soil type, followed by water regime. The evaluated biotic factors showed minor effect on the diversity and composition of endophytic communities. Rice plants cultivated in K soil (a neutral-pH soil) showed higher richness and were extensively colonized by *Pseudomonas oryzihabitans* and *Rhizobium radiobacter*, whereas plants cultivated in V soil, an acid soil, favoured the growth of *Enterobacter*-like strain REICA_082 and *Dyella ginsengisoli*. Members of these bacteria have been isolated from seeds and/or the phytosphere of various plants [11], [18], [30], [50], suggesting that they might have a long history of association with diverse host plants. Occasionally, commensalism might come into play, e.g. the plant-associated *Rhizobium radiobacter* (formerly *Agrobacterium tumefaciens*) is the causal agent of crown gall in dicotyledons, however it showed limited pathogenicity towards monocotyledons [51]. The recently-described *Dyella ginsengisoli* has originally been isolated from a ginseng field in South Korea [52]. *Dyella ginsengisoli* strain ATSB10, containing ACC deaminase and with the ability to solubilise inorganic phosphate and to produce *β*-1,3 glucanase, has been reported to increase the root length of canola seedlings by 145% [50]. The relationship of *Dyella ginsengisoli* with rice plants is unknown and this study is the first documentation that they may be associated.

In summary, seeds from rice plants harbour a great diversity of bacteria that, in response to the plant physiological status, can become competent endophytes. Some organisms might even spread out into rhizosphere and surrounding soil, therefore directly interacting with soil microbial communities [53]. Furthermore, due to their metabolic versatility, seed-borne bacterial endophytes might also increase the fitness of plants, giving the host a competitive advantage over other (indigenous) plant communities [54] and thus might affect whole-ecosystem functioning [55]. Our data suggest that under reduced habitat complexity, this assumption may be met. It remains an open question whether seed-borne endophytes are selected by the host to increase the fitness of the next generations of seeds or whether bacterial endophytes use seeds as vector for dissemination and colonization of new environments.

## Materials and Methods

### Assessment of endophytic communities from seed endosphere

Rice (*Oryza sativa* L.) seed and five-day-old seedlings from two consecutive generations were analysed. Rice seeds from cultivar APO were obtained from International Rice Research Institute (IRRI, Los Bas, Philippines) and used for seed multiplication in greenhouse conditions at the University of Groningen, Netherlands. Seeds collected from IRRI and Groningen are referred to as first and second generations, respectively. Bacterial communities of the rice seed endosphere from both generations were assessed by culture-dependent and -independent approaches. Under aseptic conditions, the hulls were removed from the rice seeds (1 g) with sterilized forceps and immediately subjected to surface-sterilization with a solution (50 ml) containing 0.12% sodium hypochlorite (NaClO), salts (0.1 and 3% sodium carbonate and sodium chloride, respectively) and 0.15% sodium hydroxide [56] at 30°C for 25 min in orbital shaking (200 rpm). The sterilization procedure was followed by a washing step to remove surface-adhered NaClO in 50 ml 2% sodium thiosulfate [57]. This procedure was repeated twice at 30°C for 10 min under orbital shaking (200 rpm) before the seeds were subjected to rehydration for 1 h at room temperature in 100 ml autoclaved demineralised (demi-)water. In addition, to assess the endophytic communities from early seedling development, 15 surface-sterilized rice seeds from both generations were incubated on R2A medium (DB - Difco) for five days at 28°C and then used to extract DNA from shoot, root and the remainder of the seed tissues.

Endophytic bacterial cells from surface-sterilized seeds and seedlings were released by disrupting the plant tissues with a soft-headed hammer as described [58]. The homogenates (100 µl) were used for serial tenfold dilutions, which were plated onto R2A, after which plates were incubated for one week at 28°C. In addition, homogenates (1 ml) were used for DNA extraction following the protocol described by Hurek et al. [56]. For each 100 mg of plant material, 1.2 ml cell lysis solution was used, while phenol∶chloroform (1∶1 v/v) was used for deproteinization. The concentration and quality of the extracted DNA were assessed by electrophoresis in 1% agarose gels, followed by staining with ethidium bromide and visualization under UV light.

### Dynamics of rice endophytes

Surface-sterilized rice seeds from the second generation were used to assessed the endophytic bacterial communities from root and shoot endosphere at three and five weeks after seed germination. The plants originating from the germinated seeds were cultivated in two soil types, i.e. Kollumerwaard – K, a clay loam soil with neutral pH (chemical characteristics: pH based on CaCl_2_ 7.3; total carbon 27.2 g kg^−1^; organic matter 40.3 g kg^−1^; dissolved organic matter 86.4 mg kg^−1^; total nitrogen 1.67 g kg^−1^; nitrate content 170.12 mg kg^−1^; and ammonium content 6.37 mg kg^−1^, soil collected from Groningen, The Netherlands) and Valthermond – V, a loamy sand soil with low pH (chemical characteristics: pH based on CaCl_2_ 4.5; total carbon 17.8 g kg^−1^; organic matter 29.2 g kg^−1^; dissolved organic matter 60.8 mg kg^−1^; total nitrogen 1.28 g kg^−1^; nitrate content 123.19 mg kg^−1^; and ammonium content 10.8 mg kg^−1^, soil collected from Drenthe, The Netherlands). Both soils were sterilized by applying gamma radiation (minimum 25 kGy, Isotron, Netherlands) and 500 g was aseptically transferred to polyester pots. Sterility of the soil was confirmed by plating, as soil suspensions prepared did not show any colony growth up to 15 days after being plated on R2A medium. Moreover, very faint (residual) bands were observed in PCR-DGGE profiles prepared with soil-extracted DNA.

For the experiment, both soils were watered to a final volume of 70% water holding capacity with filter-sterilized (0.2 µm) 25%-strength Hoagland's nutrient solution [59]. Five-day-old rice seedlings absent of visible microbial outgrowth on R2A medium (at 28°C), were individually transferred to sterile soils. Six replicates for each treatment were used. Rice plants were cultivated in the greenhouse using a day/night cycle of 16/8 h and 25/18°C for light and temperature, respectively. Soil water was replenished daily to holding capacity with freshly prepared filter-sterilized 25%-strength Hoagland's nutrient solution. At weeks three and five, plants were harvested and the bacterial communities in the root and shoot tissues were assessed by PCR-DGGE. Individual rice plants were harvested and roots were carefully washed under running tap water for the removal of adhering soil particles. Root and shoot tissues were segmented with a sterile scalpel and treated as individual sources of endophytes. The surface sterilization procedure was performed in 20-ml tubes filled with 10 ml sterilization solution by exposing rice tissues for 2 min in NaClO solution and manually vortexed at room temperature as described above. Endophytic bacterial DNA was extracted as described above.

### Invasion assay

The invasion assay consisted of rice plants cultivated in the greenhouse and subjected to different abiotic and biotic treatments. Surface-sterilized rice seeds from second generation were cultivated in two soil types, i.e. K and V, subjected to two water regimes, i.e. unflooded and flooded, and exposed to three bacterial inoculum densities (BID), i.e., low-, high- and un-inoculated (10^4^ and 10^7^ bacterial cells g^−1^ soil, respectively). To obtain an ‘artificial’ community, we used a selection of 15 previously-isolated bacteria, that resembles the community composition found in the root endosphere of mature rice plants [20], i.e. *Enterobacter* sp. strains REICA_082, REICA_112, REICA_142, *Pseudomonas* sp. REICA_175, *Klebsiella* sp. REICA_034, *Aeromonas* sp. REICA_106 and REICA_164, *Herbaspirillum* sp. REICA_064, *Shewanella* sp. REICA_181, *Exiguobacterium* sp. REICA_016, *Micrococcus* sp. REICA_095, Alphaproteobacterium sp. REICA_149 and *Mycobacterium* sp. REICA_128. In addition three presumably competent endophytes were used as controls, i.e. *Pseudomonas protegens* CHA0^T^ [49], *Pseudomonas putida* P9 [60] and *Burkholderia phytofirmans* RG44-4 [61]. Therefore we investigated which bacterium could invade the plant from soil. Each strain was grown separately in R2A broth aerobically at 28°C with shaking (200 rpm). Bacterial cells were harvested in the exponential growth phase by centrifugation and washed twice with sterile PBS buffer. Bacterial cells of each inoculum were combined with their respective amount of cells needed to achieve the final BID. The BID of each treatment was further confirmed using dilution plating on R2A medium. The mixed bacterial cells were diluted in filter-sterilized (0.2 µm) 25% Hoagland's nutrient solution, and added to the soil, establishing 70% of water holding capacity of each soil. Filter-sterilized 25% Hoagland's nutrient solution was used in control treatment (uninoculated). Inoculated and uninoculated soils (500 g pot^−1^) were covered with aluminium foil and incubated in the greenhouse for one week, for the establishment of the bacterial communities, prior to the placement of five-day old rice seedlings. One seedling per pot and six replicates per treatment were used. Rice plants were then further cultivated in the greenhouse under the aforementioned conditions. At week three, after tiller formation, plants exposed to low- and high-BID were subjected to flooding. At week five, the plants were harvested and the bacterial communities in soil free of roots (denoted bulk soil), rhizosphere soil, the root and shoot tissues were assessed by PCR-DGGE. Individual rice plants were harvested and root-adhering soil particles were removed with a forceps and stored. The bacterial endophytic community of root and shoot tissues were assessed as described above. DNA from bulk and rhizosphere soils were also extracted with the protocol described for seed samples, however DNA from these microhabitats were further purified (twice) using the Wizard DNA clean-up system (Promega).

### PCR-DGGE and ordination analyses

For PCR-DGGE analysis, the Chelius-Triplett nested PCR system (799F-1492R followed by 968F-1401R) was the most efficient approach to detect rice endophytic bacteria [62]. DNA amplification conditions and PCR-DGGE analyses were performed as described previously [58]. The denaturing gradient gel was casted with a gradient of 40–55% denaturant (100% denaturant contained 7 M urea and 40% formamide) in a PhorU-2 apparatus, (Ingeny, Goes, Netherlands). The amplicons (150 ng) from each treatment with six replicates were loaded side-by-side in the same gradient gel and were cross-compared. Reference markers containing equal amounts of DNA extracted from the inoculated strains were loaded at both edges and among treatments for normalization purposes. After the run, gels were stained with SYBR gold (Molecular Probes, Leiden, Netherlands) and the DGGE patterns were made visible by illumination with UV. The profiles were digitized using a digital camera and stored as TIFF files.

All PCR-DGGE profiles were analysed using GelCompar II v 4.06 (Applied Maths, Sint-Martens-Latem, Belgium) as described previously [58]. Relative band intensity from each PCR-DGGE profile was exported into matrix. This data combined with the biotic and abiotic factors (assigned as nominal environmental variables) were used to generated the biplot ordination diagrams by computing the redundancy analysis (RDA) from the package software CANOCO (Biometrics, PRI, Netherlands).

### Isolates and PCR-DGGE bands identification

Rice seed endophytes were isolated using R2A at 28°C and replicated on the same medium to obtain pure cultures. Single colonies were used for identification by sequencing the partial 16S rRNA gene as described [63]. For this, the reverse primer 1401R was used in the sequencing reaction. In addition, dominant bands from generated PCR-DGGE profiles were selected for identification. Following excision, band DNA was extracted by incubating the polyacrylamide gel in 50 µl sterile TAE buffer solution for two days at 4°C. From the homogenate, 2 µl was used as DNA template for PCR-DGGE re-amplification. PCR-DGGE bands with identical motility compared with the original PCR-DGGE pattern were subjected to identification by sequencing with reverse primer 1401R. Furthermore, 16S rRNA gene amplicons of rice seed endophyte strains were subjected to PCR-DGGE analysis and PCR-DGGE bands with identical denaturation motility were tentatively assigned to strains. The sequences obtained from this study were assigned to bacterial species by BlastN against NCBI nucleotide database considering only type strains as reference strains. In addition, we compared the generated sequences to publicly available seed-associated (EU741000-EU741045), [5], [9], [19], rice-associated [20], [21], [64] and rice paddy soil bacterial sequences (FJ266313-FJ266342), [27], [65]. The sequences obtained from the excised PCR-DGGE bands and the partial 16S rRNA gene from strains were deposited in the GenBank under the accession numbers JN110430 to JN110462.

## Supporting Information

Figure S1 **PCR-DGGE profiles of shoot and root endosphere bacterial community of rice cultivated in Kollumerwaard soil**. PCR-DGGE profiles of shoot A) and root B) endosphere community of rice plants cultivated in K soil. Rice plants were subjected to unflooded and flooded regimes and exposed to low-, high- and un-inoculated treatments. Six replicates per treatments are shown. Arrow heads indicate identified communities (see Table 1 and 2).
(TIF)

Figure S2 **PCR-DGGE profiles of shoot and root endosphere bacterial community of rice cultivated in Valthermond soil.** PCR-DGGE profiles of shoot A) and root B) endosphere community of rice plants cultivated in V soil. Rice plants were subjected to unflooded and flooded regimes and exposed to low-, high- and un-inoculated treatments. Six replicates per treatments are shown. Arrow heads indicate identified communities (see Table 1 and 2).
(TIF)

## References

[1] Dias ACF, Costa FEC, Andreote FD, Lacava PT, Teixeira MA (2009). Isolation of micropropagated strawberry endophytic bacteria and assessment of their potential for plant growth promotion.. World J Microbiol Biotechnol.

[2] Lucero M, Barrow JR, Osuna P, Reyes I (2008). A cryptic microbial community persists within micropropagated *Bouteloua eriopoda* (Torr.) Torr. cultures.. Plant Sci.

[3] Baker KF, Smith SH (1966). Dynamics of seed transmission of plant pathogens.. Annu Rev Phytopathol.

[4] Mundt JO, Hinkle NF (1976). Bacteria within ovules and seeds.. Appl Environ Microbiol.

[5] Mano H, Tanaka F, Watanabe A, Kaga H, Okunishi S (2006). Culturable surface and endophytic bacterial flora of the maturing seeds of rice plants (*Oryza sativa*) cultivated in a paddy field.. Microbes Environ.

[6] Kaga H, Mano H, Tanaka F, Watanabe A, Kaneko S (2009). Rice seeds as sources of endophytic bacteria.. Microbes Environ.

[7] Mano H, Morisaki H (2008). Endophytic bacteria in the rice plant.. Microbes Environ.

[8] Lucero ME, Unc A, Cooke P, Dowd S, Sun S (2011). Endophyte microbiome diversity in micropropagated *Atriplex canescens* and *Atriplex torreyi* var *griffithsii*.. PLoS ONE.

[9] Johnston-Monje D, Raizada MN (2011). Conservation and diversity of seed associated endophyes in *Zea* across boundaries of evolution, ethnography and ecology.. PLoS ONE.

[10] Puente ME, Li CY, Bashan Y (2009). Endophytic bacteria in cacti seeds can improve the development of cactus seedlings.. Environ Exp Bot.

[11] Hallmann J, Berg G, Schulz BJE, Boyle CJC, Sieber TN (2006). Spectrum and population dynamics of bacterial root endophytes.. Microbial Root Endophytes.

[12] Reiter B, Pfeifer U, Schwab H, Sessitsch A (2002). Response of endophytic bacterial communities in potato plants to infection with *Erwinia carotovora* subsp *atroseptica*.. Appl Environ Microb.

[13] Hardoim PR, van Overbeek LS, van Elsas JD (2008). Properties of bacterial endophytes and their proposed role in plant growth.. Trends Microbiol.

[14] Compant S, Clement C, Sessitsch A (2010). Plant growth-promoting bacteria in the rhizo- and endosphere of plants: Their role, colonization, mechanisms involved and prospects for utilization.. Soil Biol Biochem.

[15] Glick BR, Todorovic B, Czarny J, Cheng Z, Duan J (2007). Promotion of plant growth by bacterial ACC deaminase.. Crit Rev Plant Sci.

[16] Holland MA (1997). Occam's razor applied to hormonology. Are cytokinins produced by plants?. Plant Physiol.

[17] Taghavi S, van der Lelie D, Hoffman A, Zhang YB, Walla MD (2010). Genome sequence of the plant growth promoting endophytic bacterium *Enterobacter* sp 638.. PLoS Genet.

[18] Cottyn B, Debode J, Regalado E, Mew TW, Swings J (2009). Phenotypic and genetic diversity of rice seed-associated bacteria and their role in pathogenicity and biological control.. J Appl Microbiol.

[19] Okunishi S, Sako K, Mano H, Imamura A, Morisaki H (2005). Bacterial flora of endophytes in the maturing seed of cultivated rice (*Oryza sativa*).. Microbes Environ.

[20] Hardoim PR, Sessitsch A, Reinhold-Hurek B, van Overbeek LS, van Elsas, Hardoim PR (2011). Assessment of rice root endophytes and their potential for plant growth promotion.. Bacterial endophytes of rice – their diversity, characteristics and perspectives.

[21] Mano H, Tanaka F, Nakamura C, Kaga H, Morisaki H (2007). Culturable endophytic bacterial flora of the maturing leaves and roots of rice plants (*Oryza sativa*) cultivated in a paddy field.. Microbes Environ.

[22] Hashidoko Y, Hayashi H, Hasegawa T, Purnomo E, Osaki M (2006). Frequent isolation of sphingomonads from local rice varieties and other weeds grown on acid sulfate soil in South Kalimantan, Indonesia.. Tropics.

[23] Lebuhn M, Achouak W, Schloter M, Berge O, Meier H (2000). Taxonomic characterization of *Ochrobactrum* sp isolates from soil samples and wheat roots, and description of *Ochrobactrum tritici* sp nov and *Ochrobactrum grignonense* sp nov.. Int J Syst Evol Micr.

[24] Lai WA, Kämpfer P, Arun AB, Shen FT, Huber B (2006). *Deinococcus ficus* sp nov., isolated from the rhizosphere of *Ficus religiosa* L.. Int J Syst Evol Micr.

[25] Steindler L, Bertani I, De Sordi L, Bigirimana J, Venturi V (2008). The presence, type and role of N-acyl homoserine lactone quorum sensing in fluorescent *Pseudomonas* originally isolated from rice rhizospheres are unpredictable.. FEMS Microbiol Lett.

[26] Behrendt U, Ulrich A, Schumann P, Naumann D, Suzuki K (2002). Diversity of grass-associated *Microbacteriaceae* isolated from the phyllosphere and litter layer after mulching the sward; polyphasic characterization of *Subtercola pratensis* sp nov., *Curtobacterium herbarum* sp nov and *Plantibacter flavus* gen. nov., sp nov.. Int J Syst Evol Micr.

[27] Kämpfer P, Rainey FA, Andersson MA, Lassila ELN, Ulrych U (2000). *Frigoribacterium faeni* gen. nov., sp nov., a novel psychrophilic genus of the family *Microbacteriaceae*.. Int J Syst Evol Micr.

[28] Shrestha PM, Noll M, Liesack W (2007). Phylogenetic identity, growth-response time and rRNA operon copy number of soil bacteria indicate different stages of community succession.. Environ Microbiol.

[29] Hallmann J, Quadt-Hallmann A, Mahaffee WF, Kloepper JW (1997). Bacterial endophytes in agricultural crops.. Can J Microbiol.

[30] Oehrle NW, Karr DB, Kremer RJ, Emerich DW (2000). Enhanced attachment of *Bradyrhizobium japonicum* to soybean through reduced root colonization of internally seedborne microorganisms.. Can J Microbiol.

[31] Ryan RP, Monchy S, Cardinale M, Taghavi S, Crossman L (2009). The versatility and adaptation of bacteria from the genus *Stenotrophomonas*.. Nat Rev Microbiol.

[32] Hayward AC, Fegan N, Fegan M, Stirling GR (2010). *Stenotrophomonas* and *Lysobacter*: ubiquitous plant-associated gamma-proteobacteria of developing significance in applied microbiology.. J Appl Microbiol.

[33] Mehnaz S, Kowalik T, Reynolds B, Lazarovits G (2010). Growth promoting effects of corn (*Zea mays*) bacterial isolates under greenhouse and field conditions.. Soil Biol Biochem.

[34] Idris A, Labuschagne N, Korsten L (2009). Efficacy of rhizobacteria for growth promotion in sorghum under greenhouse conditions and selected modes of action studies.. J Agr Sci.

[35] De Freitas JR, Banerjee MR, Germida JJ (1997). Phosphate-solubilizing rhizobacteria enhance the growth and yield but not phosphorus uptake of canola (*Brassica napus* L).. Biol Fertil Soils.

[36] Sturz AV, Matheson BG, Arsenault W, Kimpinski J, Christie BR (2001). Weeds as a source of plant growth promoting rhizobacteria in agricultural soils.. Can J Microbiol.

[37] van der Lelie D, Taghavi S, Monchy S, Schwender J, Miller L (2009). Poplar and its bacterial endophytes: coexistence and harmony.. Crit Rev Plant Sci.

[38] Taghavi S, Garafola C, Monchy S, Newman L, Hoffman A (2009). Genome survey and characterization of endophytic bacteria exhibiting a beneficial effect on growth and development of poplar trees.. Appl Env Microbiol.

[39] Mastretta C, Taghavi S, van der Lelie D, Alessio M, Francesca G (2009). Endophytic bacteria from seeds of *Nicotiana tabacum* can reduce cadmium phytotoxicity.. Int J Phytoremediat.

[40] Compant S, Duffy B, Nowak J, Clement C, Ait Barka E (2005). Use of plant growth-promoting bacteria for biocontrol of plant diseases: principles, mechanisms of action, and future prospects.. Appl Environ Microb.

[41] Weyens N, van der Lelie D, Taghavi S, Newman L, Vangronsveld J (2009). Exploiting plant-microbe partnerships to improve biomass production and remediation.. Trends Biotechnol.

[42] Kageyama K, Nelson EB (2003). Differential inactivation of seed exudate stimulation of *Pythium ultimum* sporangium germination by *Enterobacter cloacae* influences biological control efficacy on different plant species.. Appl Environ Microb.

[43] Roberts DP, McKenna LF, Lohrke SM, Rehner S, de Souza JT (2007). Pyruvate dehydrogenase activity is important for colonization of seeds and roots by *Enterobacter cloacae*.. Soil Biol Biochem.

[44] Patten CL, Glick BR (2002). Role of *Pseudomonas putida* indoleacetic acid in development of the host plant root system.. Appl Environ Microb.

[45] Glick BR, Jacobson CB, Schwarze MMK, Pasternak JJ (1994). 1-aminocyclopropane-1-carboxylic acid deaminase mutants of the plant-growth promoting rhizobacterium *Pseudomonas putida* GR12-2 do not stimulate canola root elongation.. Can J Microbiol.

[46] Belimov AA, Safronova VI, Sergeyeva TA, Egorova TN, Matveyeva VA (2001). Characterization of plant growth promoting rhizobacteria isolated from polluted soils and containing 1-aminocyclopropane-1-carboxylate deaminase.. Can J Microbiol.

[47] Collavino MM, Sansberro PA, Mroginski LA, Aguilar OM (2010). Comparison of *in vitro* solubilization activity of diverse phosphate-solubilizing bacteria native to acid soil and their ability to promote *Phaseolus vulgaris* growth.. Biol Fert Soils.

[48] Loaces I, Ferrando L, Scavino AF (2011). Dynamics, diversity and function of endophytic siderophore-producing bacteria in rice.. Microb Ecol.

[49] Ramette A, Frapolli M, Sauxb MF, Gruffaz C, Meyer J-M (2011). *Pseudomonas protegens* sp. nov., widespread plant protecting bacteria producing the biocontrol compounds 2,4 diacetylphloroglucinol and pyoluteorin.. Syst Appl Microbiol.

[50] Anandham R, Gandhi PI, Madhaiyan M, Sa T (2008). Potential plant growth promoting traits and bioacidulation of rock phosphate by thiosulfate oxidizing bacteria isolated from crop plants.. J Basic Microb.

[51] De Cleene M (1985). The susceptibility of monocotyledons to *Agrobacterium tumefaciens*.. J Phytopathol.

[52] Jung HM, Ten LN, Kim KH, An DS, Im WT (2009). *Dyella ginsengisoli* sp nov., isolated from soil of a ginseng field in South Korea.. Int J Syst Evol Micr.

[53] Raaijmakers JM, Paulitz TC, Steinberg C, Alabouvette C, Moënne-Loccoz Y (2009). The rhizosphere: a playground and battlefield for soilborne pathogens and beneficial microorganisms.. Plant Soil.

[54] Klironomos JN (2002). Feedback with soil biota contributes to plant rarity and invasiveness in communities.. Nature.

[55] Himler AG, Adachi-Hagimori T, Bergen JE, Kozuch A, Kelly SE (2011). Rapid spread of a bacterial symbiont in an invasive whitefly is driven by fitness benefits and female bias.. Science.

[56] Hurek T, Reinholdhurek B, Vanmontagu M, Kellenberger E (1994). Root colonization and systemic spreading of *Azoarcus* sp. strain BH72 in grasses.. J Bacteriol.

[57] Miche L, Balandreau J (2001). Effects of rice seed surface sterilization with hypochlorite on inoculated *Burkholderia vietnamiensis*.. Appl Environ Microb.

[58] Hardoim PR, Andreote FD, Reinhold-Hurek B, Sessitsch A, van Overbeek LS (2011). Rice root-associated bacteria: insights into community structures across 10 cultivars.. FEMS Microbiol Ecol.

[59] Venema JH, Dijk BE, Bax JM, van Hasselt PR, Elzenga JTM (2008). Grafting tomato (*Solanum lycopersicum*) onto the rootstock of a high-altitude accession of *Solanum habrochaites* improves suboptimal-temperature tolerance.. Environ Exp Bot.

[60] Andreote FD, de Araujo WL, de Azevedo JL, van Elsas JD, da Rocha UN (2009). Endophytic colonization of potato (*Solanum tuberosum* L.) by a novel competent bacterial endophyte, *Pseudomonas putida* strain P9, and its effect on associated bacterial communities.. Appl Environ Microb.

[61] Sessitsch A, Coenye T, Sturz AV, Vandamme P, Ait Barka E (2005). *Burkholderia phytofirmans* sp. nov., a novel plant-associated bacterium with plant-beneficial properties.. Int J Syst Evol Microbiol.

[62] Chelius MK, Triplett EW (2001). The diversity of archaea and bacteria in association with the roots of *Zea mays* L.. Microb Ecol.

[63] Stevens P, van Elsas JD (2010). Genetic and phenotypic diversity of *Ralstonia solanacearum* biovar 2 strains obtained from Dutch waterways.. Anton Leeuw Int J.

[64] Sun L, Qiu FB, Zhang XX, Dai X, Dong XZ (2008). Endophytic bacterial diversity in rice (*Oryza sativa* L.) roots estimated by 16S rDNA sequence analysis.. Microb Ecol.

[65] Cuong ND, Nicolaisen MH, Sørensen J, Olsson S (2011). Hyphae-colonizing *Burkholderia* sp.—a new source of biological control agents against sheath blight disease (*Rhizoctonia solani* AG1-IA) in rice.. Microb Ecol.

