# Supplementary figures and images for: Dynamics of Seed-Borne Rice Endophytes on Early Plant Growth Stages
Source: PLoS One. 2012 Feb 17;7(2):e30438. doi: 10.1371/journal.pone.0030438 (PMC3281832; doi:10.1371/journal.pone.0030438)

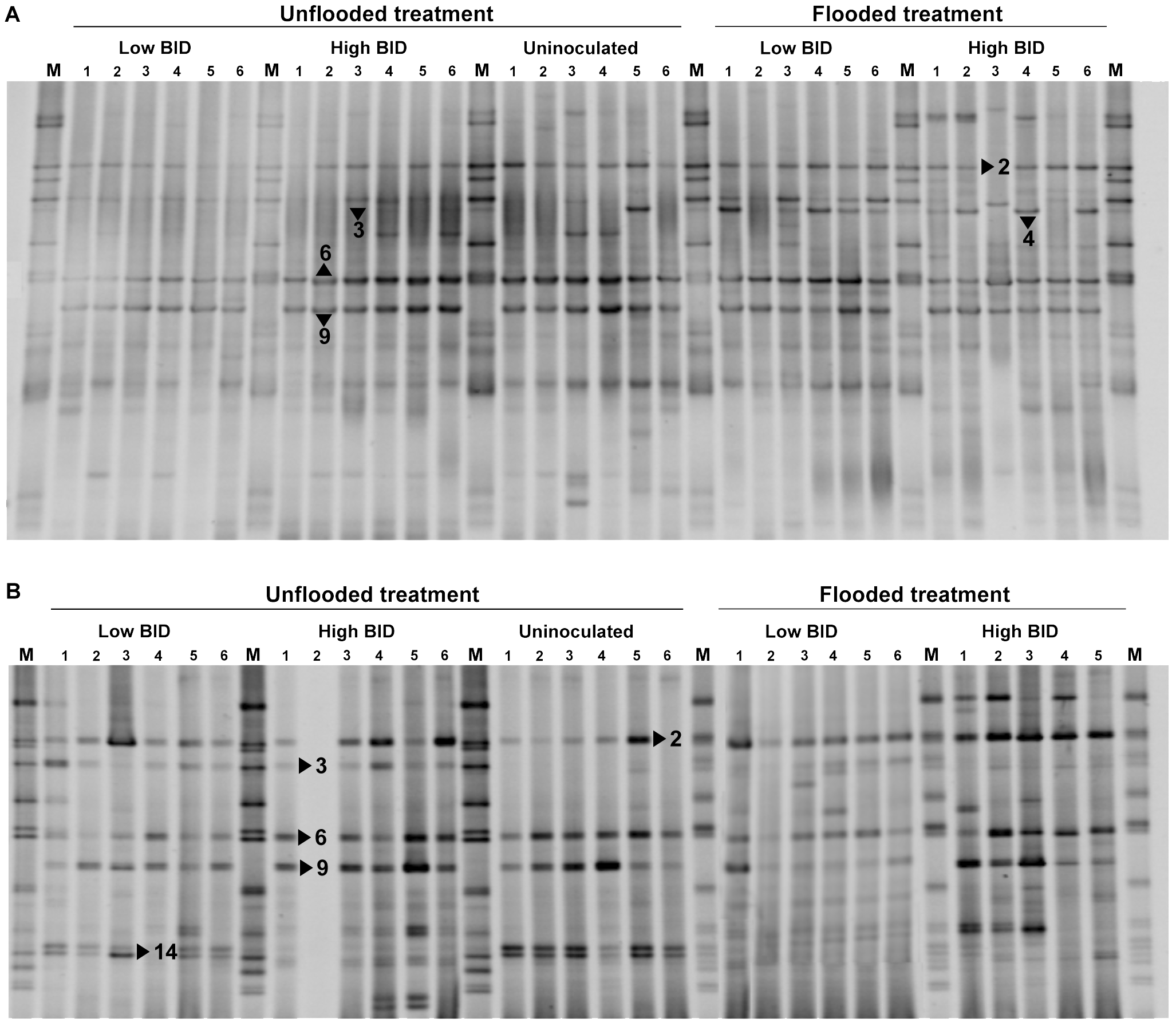

Supplement: Figure S1 — PCR-DGGE profiles of shoot and root endosphere bacterial community of rice cultivated in Kollumerwaard soil. PCR-DGGE profiles of shoot A) and root B) endosphere community of rice plants cultivated in K soil. Rice plants were subjected to unflooded and flooded regimes and exposed to low-, high- and un-inoculated treatments. Six replicates per treatments are shown. Arrow heads indicate identified communities (see Table 1 and 2). (TIF) [file pone.0030438.s001.tif]

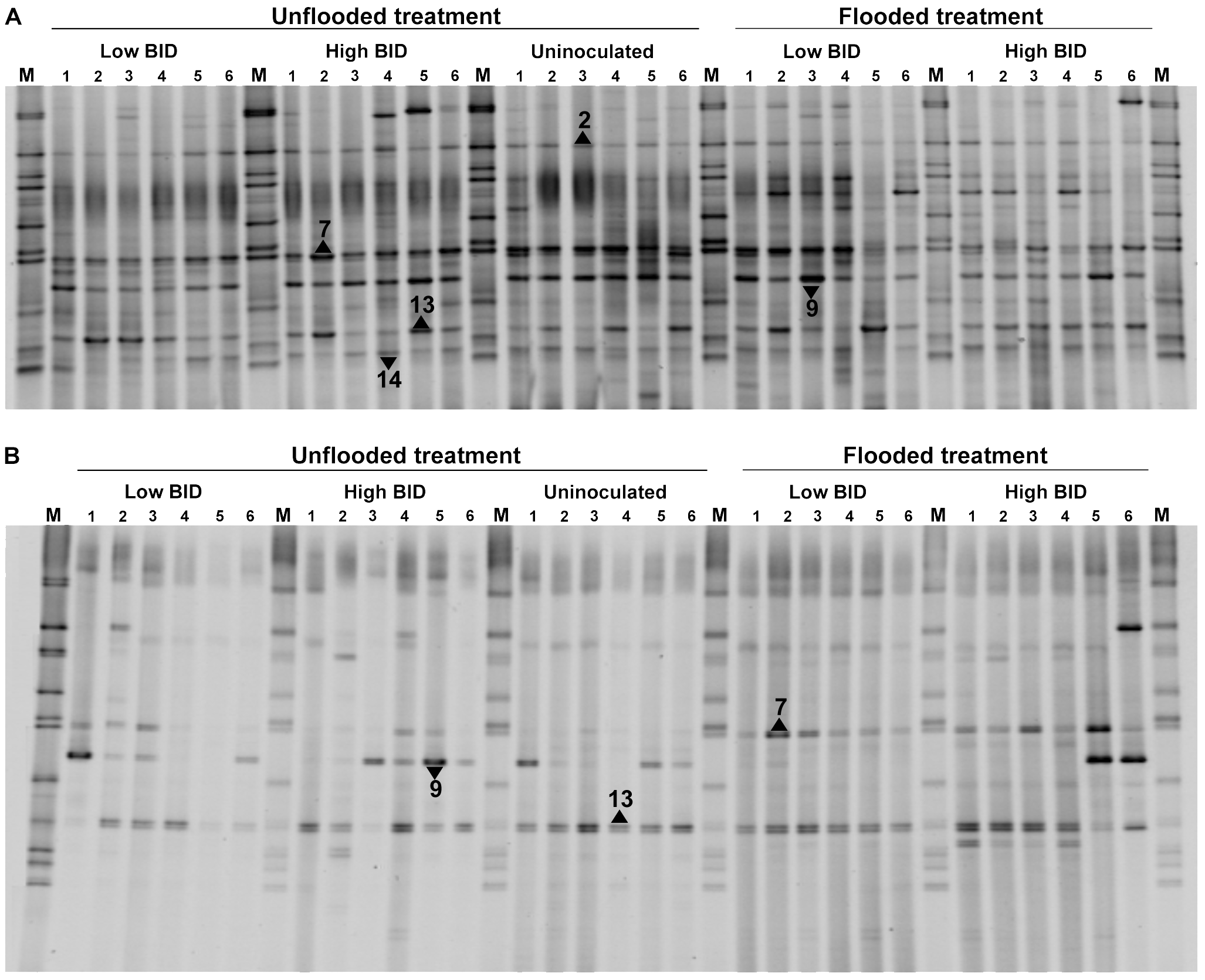

Supplement: Figure S2 — PCR-DGGE profiles of shoot and root endosphere bacterial community of rice cultivated in Valthermond soil. PCR-DGGE profiles of shoot A) and root B) endosphere community of rice plants cultivated in V soil. Rice plants were subjected to unflooded and flooded regimes and exposed to low-, high- and un-inoculated treatments. Six replicates per treatments are shown. Arrow heads indicate identified communities (see Table 1 and 2). (TIF) [file pone.0030438.s002.tif]
